# Implementing Wearable Sensors for Clinical Application at a Surgical Ward: Points to Consider before Starting

**DOI:** 10.3390/s23156736

**Published:** 2023-07-27

**Authors:** Rianne van Melzen, Marjolein E. Haveman, Richte C. L. Schuurmann, Michel M. R. F. Struys, Jean-Paul P. M. de Vries

**Affiliations:** 1 Division of Vascular Surgery, Department of Surgery, University Medical Center Groningen, University of Groningen, 9713 GZ Groningen, The Netherlands; r.c.l.schuurmann@umcg.nl (R.C.L.S.); j.p.p.m.de.vries@umcg.nl (J.-P.P.M.d.V.); 2 Department of Anesthesiology, University Medical Center Groningen, University of Groningen, 9713 GZ Groningen, The Netherlands; m.e.haveman@umcg.nl (M.E.H.); m.m.r.f.struys@umcg.nl (M.M.R.F.S.)

**Keywords:** wearable sensor, technology, vital signs, surgical ward, clinical application, continuous monitoring

## Abstract

Incorporating technology into healthcare processes is necessary to ensure the availability of high-quality care in the future. Wearable sensors are an example of such technology that could decrease workload, enable early detection of patient deterioration, and support clinical decision making by healthcare professionals. These sensors unlock continuous monitoring of vital signs, such as heart rate, respiration rate, blood oxygen saturation, temperature, and physical activity. However, broad and successful application of wearable sensors on the surgical ward is currently lacking. This may be related to the complexity, especially when it comes to replacing manual measurements by healthcare professionals. This report provides practical guidance to support peers before starting with the clinical application of wearable sensors in the surgical ward. For this purpose, the Non-Adoption, Abandonment, Scale-up, Spread, and Sustainability (NASSS) framework of technology adoption and innovations in healthcare organizations is used, combining existing literature and our own experience in this field over the past years. Specifically, the relevant topics are discussed per domain, and key lessons are subsequently summarized.

## 1. Introduction

The World Health Organization forecasts increasing global demand for healthcare and social care personnel by 2030 [1]. Additionally, the COVID-19 pandemic has exacerbated the nurse shortage globally [2]. Incorporating technology into healthcare processes is necessary to continue the provision of high-quality care in the future, especially with the increase of aged, multimorbid, and frail patients. An example of a patient population where this applies is surgical patients who are at high risk of postoperative complications [3].

In the postoperative phase, patients are classically referred from high-care units, such as the intensive care unit (ICU), to low-care settings, such as the surgical ward. In high- or medium-care units, patients’ vital signs are generally continuously monitored, sometimes with invasive means. This level of care and monitoring demands a high nurse-to-patient ratio, and, therefore, patients are transferred to the ward as soon as possible when the risks of acute events are low enough. On the ward, nurses typically measure vital signs manually every 4 to 8 h. Scoring systems, such as the Modified Early Warning Score (MEWS) [3], are calculated based on nurse observations to identify the risk of deterioration and, eventually, to scale up the level of care. However, manual intermittent monitoring by nurses could cause delays in the detection of postoperative deterioration, is time consuming, and is not always accurate or registered completely [4].

Conversely, technological developments enable continuous wireless monitoring of vital signs and physical activity of patients with wearable sensors. Wearable sensors can unlock vital signs mainly by photoplethysmography (PPG), an optical technique for blood volume variation measurements, or electrocardiography (ECG). Heart rate is the most commonly measured vital sign and is often measured accurately by both techniques [5,6,7,8]. Respiration rate can be computed from PPG or ECG signals by algorithms based on amplitude and frequency modulation, although, in general, the performance of these algorithms is better based on ECG than on PPG [9]. Nevertheless, PPG allows the measurement of blood oxygen saturation (SpO_2_) by pulse oximetry and noninvasive calculation of blood pressure by pulse wave transit time and pulse wave analysis [10]. Furthermore, the (skin) temperature can be derived from negative temperature coefficient thermistors and physical activity from accelerometry. The market is still evolving because ideal wearable sensors must be able to measure reliably, be easy to wear (lightweight) and easy to use, be low in cost, have a long battery life, and enable the patient to be ambulant [11].

Integrating wearable sensors in clinical practice, specifically in the surgical ward, could be beneficial in several ways. First, the workload of health care professionals could be reduced by replacing intermittent manual measurements of vital signs with continuous vital sign monitoring. Second, continuous monitoring of vital signs and physical activity may assist healthcare professionals in the early detection of patient deterioration. Third, wearable sensors may contribute to the evaluation of patient recovery and support healthcare professionals in decision making [12].

Although the use of wearable sensors in the surgical ward has great potential, research on the subject has mostly been restricted to limited sample sizes. Recently, Leenen et al. reviewed the validity, feasibility, and clinical and cost outcomes of available sensors for continuous monitoring of at least two vital signs in hospitalized patients [13]. Of the 13 included sensors from 10 different manufacturers in their review, only three comprised the wearable sensors that were used on a general ward. They concluded that research on this topic is mainly still in the validation and feasibility phase [13]. Moreover, a recent meta-analysis evaluated the effect of wearable sensors on the detection of deterioration and related clinical outcome metrics in hospitalized patients [14]. The meta-analysis included only seven studies with a moderate level of evidence. In summary, wearable sensors are not yet widely used in clinical practice in the surgical ward, and the aforementioned expectations have not been supported by strong evidence. 

The current lack of successful application of wearable sensors on the surgical ward might be related to their complexity and the consequences for scalability in clinical practice, especially when it comes to replacing actions performed by healthcare professionals. Our research group (consisting of technical physicians, anesthesiologists, surgeons, a clinical epidemiologist, and a nurse scientist) has performed several studies with wearable sensors on the surgical ward in which relevant lessons were learned. In this report, we aim to provide practical guidance to support peers in getting started with monitoring patients using wearable sensors in a surgical ward. 

## 2. Materials and Methods

The Non-Adoption, Abandonment, Scale-up, Spread, and Sustainability (NASSS) framework [15] offers a systematic evidence-based approach to cover the wide range of aspects involved in the use of wearable sensors in a surgical ward. The NASSS framework is developed for predicting and evaluating the success of technology-supported healthcare and social care programs. This framework supports implementation teams to identify and address the key challenges in different domains and the interactions between them. It consists of seven domains classified by different levels of complexity: (1) the condition, (2) the technology, (3) the value proposition, (4) the adopter system (staff, patient, caregivers), (5) the organization, (6) the wider system, and (7) embedding and adaptation over time. In relation to our expertise, the emphasis of this report is on the first four domains of the framework. Relevant topics from existing literature and our experiences are discussed per domain, and the key lessons per domain are summarized. These lessons are intended for peers to consider before starting with the clinical application of wearable sensors on the surgical ward and can be used to guide conversations and generate research ideas. The domains of the NASSS framework will be illustrated with data from a patient who was continuously monitored by two different wearable sensors (one ECG-based (VitalPatch, MediBioSense, Doncaster, UK) and one PPG-based (Radius PPG and Radius T, Masimo, Irvine, CA, USA)) in the surgical ward in an ongoing observational study at the University Medical Center Groningen.

## 3. Results

### 3.1. Condition

This domain of the NASSS framework comprises the clinical context, comorbidities, and sociocultural aspects of patients for which wearable sensors will be used [15]. It reflects the importance of identifying potential end users. To be able to implement wearable sensors in surgical wards, the types of patient for whom continuous monitoring can be of added value needs to be specified. For example, patients undergoing major abdominal surgery have complication rates of up to 44% [16]. Approximately 60% of these complications, such as respiratory failure, surgery-associated complications, pulmonary embolus, myocardial infarction, and congestive heart failure, occur within 3 postoperative days [17]. This type of information can help determine when the use of wearable sensors will be most effective for this specific population. In this example, the use of wearable sensors for early detection of postoperative complications may be most relevant in the first 3 days after surgery. 

**Figure 1:**
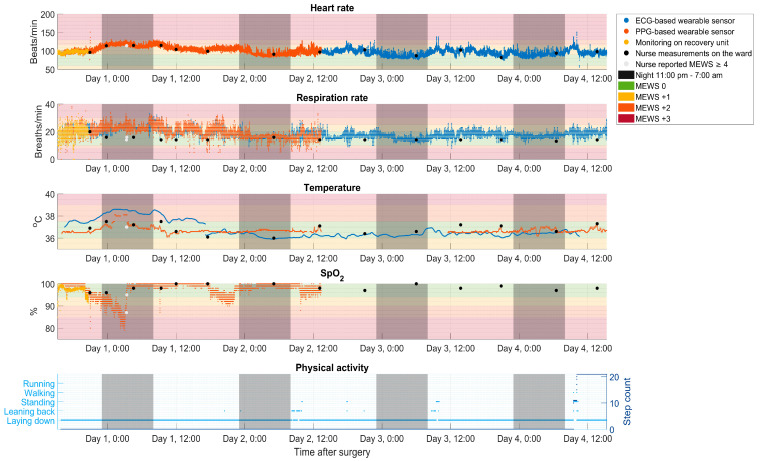
Vital signs and physical activity measurements of a 20-year-old female patient during the first four postoperative days after open fixation of the thoracic spine following multitrauma. Top to bottom: raw measurements of four vital signs (heart rate, respiration rate, temperature, and blood oxygen saturation [SpO_2_]) measured by two wearable sensors, an ECG-based sensor (blue) and a PPG-based sensor (orange), by a bedside monitor on the recovery unit (yellow) and by nurses on the surgical ward (black dots). Colored areas represent the limits for points in the Modified Early Warning Score (MEWS) per vital sign. An elevated MEWS (≥4) requires more frequent patient surveillance by hospital protocol (light grey dots). Bottom: physical activity measured as a type of activity and step count by the ECG-based sensor in light blue and dark blue, respectively. Nighttime (11:00 p.m. to 07:00 a.m.) is depicted by grey areas. Data were extracted from an ongoing project in the University Medical Center Groningen (approved by the medical ethical committee, METc 2021/440) for which the patient signed informed consent.

Before wearable sensors are implemented, the first step is to define the clinical purpose to accomplish customized use and to prevent the unnecessary use of time and resources. In addition, this knowledge can support healthcare professionals and policymakers in adopting the use of wearable sensors. Postoperative continuous monitoring of vital signs could also be of added value for earlier detection and possible prevention of hypotension, hypoxemia, apnea, or atrium fibrillation compared to intermittent monitoring at the ward [18]. Figure 1 shows an example of vital signs and physical activity monitored for a 20-year-old female patient by two wearable sensors during the first four postoperative days after thoracic surgery following multitrauma. During the first postoperative night after discharge from the recovery unit, SpO_2_ decreased to 87%, as measured by the nurse at 03:19 a.m., which could already be observed with continuous monitoring shortly after midnight. 

Postoperative hypoxia (SpO_2_ < 90%) occurs frequently, and prolonged hypoxemia may result in arrhythmias, myocardial ischemia, and cognitive dysfunction [19]. Furthermore, prolonged desaturation may increase the risk of surgical site infections. However, monitoring SpO_2_ to target normoxemia in surgical patients with wearable sensors could prevent late detection of hypoxemia.

Previous studies on wearable sensors for continuous monitoring in the surgical ward have been mainly performed in postoperative patients [6,7,8]. However, which patient-specific characteristics influence the applicability of this technology is still unknown. Studies with wearable sensors in surgical patients are prone to selection bias, where participants often have a higher education [20] and are younger [21] compared with nonparticipants. One study in older surgical patients (aged 65 years or older) reported that nonparticipants were more often women, unmarried, living alone, and had a higher American Society of Anesthesiologists Physical Status Classification and more polypharmacy [21]. Generalizability to all surgical patients can therefore be a challenge. We advise specifying the patient characteristics of the population for which the use of wearable sensors should be feasible in an early stage. 

In addition to patient characteristics, other patient-related factors play an important role in the ability to wear a sensor in the surgical ward. In a recent study by our research group, surgical patients wore a wearable sensor preoperatively at home until 30 days postoperatively. We found several main barriers to participation in and completion of the study, such as surgery-related mental burden, patients’ frailty, cognitive state, and low health literacy [22]. When the active contribution of patients is required, socioeconomic status and degree of education affect the acceptance and usability [23]. Overall, the vulnerability of the patient must be taken into account. For example, in the case of dementia or delirium, application of wires and plasters to the body should be minimized given the risk of the patient removing it. In such cases, professionals should evaluate the burden on the patient versus the benefits of continuous data from the wearable sensor. Although high generalizability is desired, not all patients may be able to be monitored, especially vulnerable or restless patients after surgery.

### 3.2. Technology

The technology domain addresses the features of and measured parameters by wearable sensors. This domain also includes the knowledge needed to use this technology and the extent to which adjustments are still needed within the organization for its daily use [15]. The potential of wearable sensors for monitoring vital signs increases as a result of the development of smaller sensors with improving battery capacity and a growing number of measured parameters. Regarding the choice of wearable sensors, the following items should be considered. 

First, professionals should define which parameters are most relevant to be monitored by a wearable sensor based on the clinical purpose. Monitoring potential tachycardia in the early postoperative period or detection of respiratory insufficiencies over a longer period requires different parameters to be monitored. For example, postoperative atrium fibrillation occurs at a rate of up to 6.5% in patients after general surgery when continuously monitored at the ward, mainly on days 2 and 3, although this was not associated with prior deviation of vital signs [24]. This may require close monitoring of the heart rate (ECG). In our example, desaturation, as illustrated in Figure 1, was only measured by one of the sensors. Therefore, careful selection of sensor technology in relation to ongoing pathophysiological alterations is required. 

Second, personnel should determine the desired quality of these measurements for the clinical purpose. The quality of the measured parameters should be investigated in the concerned patient population because validity and reliability of wearable sensors are often not assessed outside a controlled environment. Information about the quality of measurements by wearable sensors is essential for their use in clinical decision making because it affects the interpretation of data. In the absence of acceptable cutoff values for the reliability of wearable sensors, we reached a consensus in our research group on the acceptable mean difference between wearable sensors and reference measurements from the Bland–Altman analyses, as shown in Table 1. These values are based on the MEWS and on prior literature [6,8]. Nevertheless, the quality of measurements should be in reasonable proportion to the clinical purpose, for which the trending ability of the sensor may be as important.

In addition to the first and second items regarding the measured parameters, the following literature findings concerning respiration rate, SpO_2_, and temperature measurements by wearable sensors should be taken into account. Compared with heart rate, respiration rate and SpO_2_ measurements are more influenced by movement. Stam et al. demonstrated that movement artifacts easily corrupted respiration rate measurements with a wrist-worn sensor based on PPG [25], which led to the exclusion of 66% of the measurements due to a low quality index. Similarly, we derived approximately 30% fewer SpO_2_ values compared with heart rate and respiration rate measurements in a clinical validation study with an arm-worn sensor based on PPG in postoperative patients in the surgical ward [7]. Data availability of both parameters also decreased during physical activity in a validation study of wearable sensors during daily life activities in volunteers [5]. The position of the sensor on the body and differences in measurement techniques of wearable sensors might also play a role. Specifically, respiration rate measurements improve with sensor proximity to the chest [26], and respiration rate measurements with a patch on the chest based on ECG may be more accurate compared with an arm-worn sensor based on PPG [5]. The example in Figure 1 shows large variability in respiration rate measurements of both sensor types. SpO_2_ is commonly measured at the fingertip, which enables transmission mode PPG with higher perfusion in contrast to more convenient measurement sites that require reflection mode PPG, such as the upper arm [27]. Furthermore, only a few wearable sensors enable SpO_2_ measurements [18].

Regarding temperature measurements, there are a number of things to consider. In one of our previous studies, visual inspection of the continuous data from a wearable sensor showed increasing temperature values up to 60 min after sensor placement [7]. This warming-up period needs to be taken into account during interpretation. Additionally, some manufacturers use calibration with a clinically used thermometer once and/or repeatedly to guarantee certain reliability of the temperature measurements. This requires extra action from healthcare professionals. Moreover, the gold standard for temperature is rectal or bladder temperature, which is infrequently assessed in general wards and, therefore, is difficult to use for calibration or validation. Alternative temperature measurements are observer-dependent, which means the calibration will also depend on the observer. Figure 1 illustrates the moment of temperature calibration of the ECG-based sensor at postoperative day 1 around 5:00 p.m. The difference of 1 degree makes the accuracy of the previous measurements uncertain. Most validation studies on the temperature measured by wearable sensors are performed in comparison with nurse measurements and report mean differences higher than the ±0.5° Celsius as stated in Table 1 [18]. Lastly, caution is needed when interpreting temperature due to potential environmental influences, such as the temperature in the room or whether the sensor is below or above the blanket. 

Third, other relevant properties of the wearable sensors may influence the measurements and implementation. Battery life can vary significantly among wearable sensors, ranging from 12 h to several days depending on the type of measurements, number of measured parameters, and sample frequency. The ECG-based sensor in the example of Figure 1 has a battery life of up to 7 days, while the PPG-based sensor lasts up to 4 days. Frequent battery charging or the need to change the sensor in case of disposable sensors requires a significant time investment for healthcare providers. In addition, not replacing the battery immediately leads to missing data. On top of that, the use of multiple disposables is costly and nonsustainable. For these reasons, the duration of monitoring needs to be chosen carefully. Clear agreements should be made with suppliers about the expiration date of disposables, the use and storage of data, and the interoperability of the wearable sensors with the existing hospital information technology systems. Additionally, in one of our studies, we experienced problems with the software that was used not being in a language understood by the healthcare professionals. Finally, professionals should consider choosing reusable wearable sensors when those are easier to use because they can be cheaper in the long run and may be more sustainable by reducing unnecessary electronic waste. Both sensors used in the example of Figure 1 were disposable and single use.

### 3.3. Value Proposition

This domain comprises the value proposition for the patient, the healthcare professional, and the healthcare organization. Knowing whether wearable sensors are worth using in the surgical ward at all is important because a mismatch frequently exists between the value for the manufacturer and for patients or healthcare professionals [15]. 

As mentioned before, the use of wearable sensors for continuous monitoring in the surgical ward is hypothesized to play a role in the early detection of deterioration and postoperative complications and by reducing the workload of healthcare professionals. However, evidence for the (cost-)effectiveness of wearable sensors is lacking [12,13]. For the patient, the added value of using wearable sensors might be a feeling of empowerment or security by high-frequency monitoring. In addition, continuous monitoring by wearable sensors might save time and money by reducing the number of unnecessary hospital visits. Even more, monitoring of clinical deterioration and early detection of postoperative complications is critical to ensure patients’ safety and well-being. 

In an earlier review by our research group, examples of relevant outcome measures common to all patients were suggested, such as hospital length of stay and recovery in terms of time to full mobilization, and well-being parameters, such as pain and mental well-being, as part of the Patient Reported Outcomes Measurements (PROMs) [12]. Early detection of deterioration in hospitalized patients by wearable sensors caused a reduction in intensive care unit admissions, rapid response teams, cardiac arrest calls, and complications [28,29]. However, a recent study found that disease severity, length of stay, and mortality of patients with unplanned intensive care unit admission was not affected by continuous vital sign monitoring on the ward [30]. In addition to early deterioration outcomes, other reported outcomes, as a result of continuous monitoring, were better pain relief and higher patient satisfaction [31]. We advise defining outcome measures for cost-benefit analyses before starting the implementation and incorporation of this outcome in future prospective trials.

For healthcare professionals, the use of wearable sensors can also be of added value. Nowadays, vital signs are intermittently measured and electronically documented by nurses during rounds in the general ward. The frequency of measurements depends on the critical illness of the patient; however, measurements are generally performed 3 to 8 times per 24 h, as illustrated in Figure 1. In the case of increased MEWS scores, medical staff will be informed. Replacing these manual measurements with wearable sensors can increase nurse satisfaction and reduce workload perception [29].

Nevertheless, there are also critical notes to mention. First, not all parameters within the MEWS can be yet measured by a single wearable sensor. For completing the MEWS, nurses also need to measure blood pressure, consciousness, worry-indicator, and urine output. Second, a continuous data set of the vital parameters can lead to disturbance by giving unnecessary alarms and may lead to overdiagnosis and overtreatment. Third, software or algorithms needed to assist in deriving, handling, and interpreting continuously monitored vital signs are lacking. Although most healthcare professionals are not used to interpreting data from continuous monitoring in general, the suitable alarm criteria are still unknown [32,33]. As a result, the main barriers to the use of wearable sensors in the surgical ward mentioned are nurse engagement and alarm burden [34]. Figure 1 illustrates that if MEWS criteria are used for individual parameters, sensor measurements would lead to a high number of alarms due to artifacts, such as on the day of surgery around 9:00 p.m. Although automated trend analyses are often mentioned for their potential added value compared to absolute alarms [35], this area is still in its infancy. Introducing healthcare professionals with expertise in this field of technology could be of added value because these technological specialists could develop implementation strategies for feedback of data from wearable sensors. For example, they could determine how often vital sign measurements are stored in the electronic health record and enable flexibility in these frequencies. The latter allows healthcare professionals to change the frequency as the risk of patient deterioration increases or decreases. 

Implementing the use of wearable sensors for patients has the potential of cost-effectiveness in hospitals. Continuous monitoring can alert the hospital that a surgical patient is deteriorating, resulting in an early return to the hospital and, perhaps, preventing unplanned readmissions or reoperations as well as unnecessary hospital visits. For wearable sensors to be added as part of usual care for patients, the department that makes acquisition decisions will need to see a formal document that explains how the benefits of wearable sensors justify the expenditures. Reimbursement options from health insurers, which can contribute to the sustainability of wearable sensors, should also be investigated early in the process. In the Netherlands, health insurers have recently agreed to reimburse the use of wearable sensors for chronic heart failure as part of the standard of care. For other applications, reimbursement still has to be arranged.

### 3.4. Adopter System

Whether patients and healthcare providers engage with and use wearable sensors depends on how easy the sensors and technology are to use, their acceptance, how much work is required by users, and the behavior of lay caregivers [15]. As elaborated in the technology acceptance model, the actual use of technology depends on perceived usefulness, and perceived ease of use will determine the actual use of technology [36]. Predicting whether technology will be used, according to the unified theory of acceptance and use of technology, also involves performance and effort expectancies, conditions that facilitate use and social influences, and is moderated by variables such as experience, sex, age, and willingness of the individual to use the technology [37]. A clinical benefit has been associated with compliance, defined as a measure of actual use, to such technology [38]. The usability of the technology is an important pillar in the adoption of a new system. The user friendliness can mostly be described from two perspectives, i.e., healthcare professionals and patients. For patients, a wearable sensor should not complicate daily life activities, such as washing or showering. The ingress protection rate indicates whether wearable sensors may or may not be exposed to water, such as the ECG- and PPG-based sensors from Figure 1, respectively. To be worn while showering, a wearable sensor needs an ingress protection rate of at least ×4. If the sensor needs to stay dry, this has unwanted consequences in terms of extra activities for the nurse and time without monitoring the patient. Thus, possibilities to change adhesive layers or plasters are recommended. In our previous experiences, some patients developed skin irritation from the plaster material [22]. In line with this, a recent review stated that most adverse device events reported for wearable sensors on the ward are skin related [39]. In general, frequent evaluation of acceptance and required workload is needed, so that the willingness to use remains high.

Furthermore, technical issues, such as a loss of connection of the wearable sensors, may occur regularly. This causes missing data, which was also the case in Figure 1 for temperature measurements between day 2 12:00 p.m. and day 3 12:00 p.m. In a recent study with wearable sensors in the general ward, about 10% of the sensors were replaced due to technical failure prematurely [40]. Another study showed that loss of connectivity occurred in 6.5% of the total monitoring time at the ward [41]. Therefore, especially in the starting phase, we advise allocating time to solve technical issues. This learning curve phenomenon [22] is challenging and requires a lot of effort and technical support to get through. Providing technological support and training for healthcare providers and for patients and their relatives will require a preinvestment in time and money. The ability for using, accepting, and investing in the work required will also hinge on the design or redesign of the hospital’s care process. Workload frequently increases when new technologies are first used, and healthcare providers and patients may need to be assigned relevant tasks according to their skills and capabilities. However, shifting work from healthcare providers to the patients, as detailed by the Burden of Treatment Theory of May [42], creates higher demands on patients, which might be rather disempowering instead of increasing self-empowerment for patients. However, healthcare professionals and patients may both benefit from support provided by a help desk or clear manuals [43]. Moreover, for clinical application, in general, feasibility and usability may be improved with the early-phase engagement of healthcare professionals and patients [43]. 

### 3.5. The Organization, The Wider System, and Embedding and Adaptation over Time

Important factors in an organization’s ability to increase the use of wearable sensors are its readiness for technological innovation and decisions on dedicated funding to support moving from a concept to reality. Aspects of the institutional and societal system include regulatory policies for technology development as well as health and legal issues [15]. Because these policies and technologies rapidly change, it is important that the organization is flexible and able to rebound. Pilot studies evaluating the feasibility and usability of continuous monitoring provide valuable information about clinical applications in a specific population; however, there is often a large gap between research and clinical application. A shared vision is needed between healthcare providers and policymakers upon which they can build and create collective guidance on what continuous monitoring technologies can and cannot execute in their institutions [15]. Furthermore, a space in the hospital could be used for device exchanges and for service and training for patients and healthcare providers.

Patients and healthcare providers are also concerned about safety and privacy issues [44,45]. Data from monitoring devices are commonly collected only within the hospital, whereas data from wearable sensors can also be acquired outside the hospital. The use of these technologies might be influenced by trust in safety and privacy aspects. It will be important for the network infrastructure that is chosen to comply with regulations on who is allowed to access data, and the contracts and Informed consents need to specify the appropriate use of data for research and development [45]. The ability to obtain support will also be desirable, ideally without substantial delay of the process, to cope with the continuously changing technology as well as legal, regulatory, and privacy issues.

The organization will need to be able to adapt to rapid changes in technology development. The technology domain suggests ideas on how to conduct this reevaluation. Depending on the actual differences in technology, the value proposition (domain 3) and adoption (domain 4) should be studied again, such as potential changes in ease of use or when different variables are monitored. Organizations are recommended to be mostly sensor independent to enable interoperability and standardized exchange of data, with which sensor suppliers need to comply, such as the MedMij criteria in the Netherlands [46]. The number of companies in the field of wearable sensor technology and software is increasing, and the organization needs to take into account that these companies can also potentially disappear from the market, which can affect the scalability and sustainability of the service.

In summary, Table 2 lists the key lessons per NASSS domain to consider before wearable sensors are used on the surgical ward, as previously discussed. 

## 4. Future Perspectives

Based on existing literature and having several years of experience in the field of wearable sensors, we applied a framework providing key lessons in this study for the clinical application of wearable sensors in the surgical ward. One of the striking conclusions is that robust evidence is lacking in all domains of the NASSS framework and that more research is needed. Although research has been performed on the reliability and validity of vital sign measurements of wearable sensors, results are largely based on small-scale observational pilot studies. Nevertheless, the type of evaluation should match the quality of technology (technology readiness level (TRL)). For example, the adoption and (cost-)effectiveness of wearable sensors on the surgical ward can only be properly assessed after full integration and adaptation of clinical processes. A framework of service readiness levels has been developed to evaluate evidence based on the maturity of digital health innovations and the stage of the decision-making process and can be used to promote scaling [47].

We learned from our experiences that the clinical application of wearable sensors on the surgical ward requires a step-wise approach. The first step focuses on the technology. First of all, professionals should assess the quality of a new wearable sensor technology in a controlled environment (technology domain; TRL 6), such as an eHealth House [12]. Subsequently, professionals need to evaluate the quality of the wearable sensor technology with respect to reference devices in the patient population (both condition and technology domains; TRL 7), for example, in the recovery unit compared to the bedside monitor.

The second step contains an assessment of the feasibility and usability of the wearable sensor in clinical practice in a prospective pilot study on the surgical ward (as part of the adopter system domain). The result of these two steps may be that a wearable sensor is not able to fulfill the criteria for a particular clinical purpose and that it is better to proceed with a different wearable sensor.

The third step is to scale up the technological and clinical integration and evaluate the adoption and effectiveness of the wearable sensor on the surgical ward (value proposition, adopter system, and healthcare organization domains). There are several reasons why randomized controlled trials may not work well for the evaluation of these outcomes. These include rapidly evolving technologies, the dependence on patient preferences and acceptability, and embedding in clinical practice [48]. In addition, performing a truly double-blind study would be very difficult. Relton et al. proposed the idea of a cohort multiple randomized controlled trial as an alternative to randomized controlled trials. A large observational cohort is recruited, from which regular outcome measurements are obtained. Then, patients in this cohort are randomly selected to receive an intervention [49]. This may be more suitable when technology develops over time. 

## 5. Conclusions

The application of wearable sensors for continuous monitoring of vital signs and physical activity in current healthcare processes in the surgical ward is complex and challenging. Although progress is being made in experience and research, full embedding in practice is still lacking. This perspective provides a practical guide to help peers before starting with the clinical application of wearable sensors on a surgical ward. 

## Figures and Tables

**Table 1:** Cutoff values of acceptable difference between wearable sensor and gold standard reference measurements.

Vital Sign	Acceptable Difference
Heart rate	±5 beats per minute
Respiration rate	±2 breaths per minute
Temperature	±0.5 °C
Blood oxygen saturation (SpO_2_)	±2%

**Table 2:** Key lessons per NASSS domain to consider before getting started with clinical application of wearable sensors on the surgical ward.

Domain	Key Lessons
Condition	Determine the clinical goal(s) Specify the characteristics of the patient population Make a trade-off between the impact of wearing the sensor for the patient and continuous monitoring for the healthcare professional versus the intended goal
Technology	Define what to monitor based on the clinical purpose Decide on (and evaluate) the appropriate measurement quality Understand the additional properties of the wearable sensor
Value proposition	Set relevant outcome measures Develop strategies for feedback of the data Prepare a business case
Adopter system	Assess the usability for end users Allocate time to solve technological problems Involve end users in the implementation
Healthcare organization Wider system Embedding and adaptation over time	Discuss the ability of data integration at an early stage Find support to comply with law and regulation Build sustainable information technology infrastructure for future developments

## Data Availability

Not applicable.
